# Intranasal Human-Recombinant Nerve Growth Factor Enhances Motor and Cognitive Function Recovery in a Child with Severe Traumatic Brain Injury

**DOI:** 10.3390/ph18020163

**Published:** 2025-01-25

**Authors:** Lorenzo Di Sarno, Lavinia Capossela, Serena Ferretti, Luigi Manni, Marzia Soligo, Susanna Staccioli, Eleonora Napoli, Riccardo Burattini, Antonio Gatto, Antonio Chiaretti

**Affiliations:** 1 Institute of Pediatrics, Fondazione Policlinico A. Gemelli IRCCS, 00168 Rome, Italy; lorenzodisarno1993@gmail.com (L.D.S.); laviniacapossela@gmail.com (L.C.); serenaferretti04@gmail.com (S.F.); antonio.gatto@policlinicogemelli.it (A.G.); 2 Istituto di Farmacologia Traslazionale, Consiglio Nazionale delle Ricerche (CNR), 00133 Rome, Italy; luigi.manni@ift.cnr.it (L.M.); marzia.soligo@ift.cnr.it (M.S.); 3 Dipartimento di Neuroriabilitazione Intensiva, Ospedale Pediatrico “Bambino Gesù”, 00050 Rome, Italy; susanna.staccioli@opbg.net (S.S.); riccardo.burattini@opbg.net (R.B.); 4 Unit of Neurorehabilitation, Department of Neurosciences, Bambino Gesù Children’s Hospital, IRCCS, 00050 Rome, Italy; eleonora.napoli@opbg.net; 5 Institute of Pediatrics, Fondazione Policlinico A. Gemelli IRCCS, Università Cattolica Sacro Cuore, 00168 Rome, Italy

**Keywords:** brain injury, children, cognitive functions, diffuse axonal injury, nerve growth factor, intranasal administration

## Abstract

**Introduction**: Traumatic brain injury (TBI) in pediatric population is responsible for significant mortality and morbidity, particularly among children aged 0–4 and young adults aged 15–24. The developing brain’s unique characteristics may increase vulnerability to injuries, potentially leading to long-term cognitive and motor deficits. Current therapeutic options for neuronal regeneration post-TBI are limited, although neurotrophins, especially nerve growth factor (NGF), show promise in enhancing recovery. NGF can mitigate excitotoxicity and promote neuroprotection, particularly by intranasal administration, which is attractive because of its non-invasive nature. **Case Presentation**: A three-year-old boy suffered from severe TBI due to a car accident, leading to multiple complications, including a basilar skull fracture and cerebral venous sinus thrombosis. Initial assessments revealed significant neurological impairments. After intensive care and rehabilitation, the child exhibited gradual improvements in consciousness and motor functions but continued to face challenges, particularly with left-sided hemiparesis. Nine months post-injury, he began intranasal administration of human-recombinant NGF (hr-NGF) as part of a clinical trial. **Discussion**: Following hr-NGF treatment, the child demonstrated notable advancements in motor function, achieving independent standing and walking. Cognitive assessments indicated improvements in various domains, including verbal comprehension and executive functioning. EEG results showed reduced epileptiform activity. These findings suggest that hr-NGF may facilitate recovery in pediatric TBI cases by enhancing both motor and cognitive outcomes. **Conclusions**: This case highlights the potential role of intranasal hr-NGF administration as a therapeutic strategy for improving neurological recovery in children with severe TBI. The positive clinical outcomes support further exploration of NGF as a viable treatment option to mitigate long-term sequelae associated with pediatric brain injuries.

## 1. Introduction

Traumatic brain injury (TBI) has a specific burden in terms of mortality and morbidity in pediatric age [1]. It poses a significant healthcare issue from the time of initial damage until many years later, when long-term sequelae may eventually appear. Some age groups have a higher risk of developing long-term neurological damage. These include children aged 0–4 years and young people aged 15–24 years [2]. In fact, a child’s developing brain is a dynamic environment with a unique cellular composition, neural circuitry and blood flow [3]. These characteristics have a significant impact on its susceptibility to insults. Although it is assumed that younger brains naturally recover better than older ones, several studies have suggested that a young brain may be more vulnerable to injury [4,5]. Damage during a critical period of development could eventually lead to cessation of function, either cognitive or motor [6]. In fact, behavioral and psychological damages are common long-term sequelae of severe TBI in pediatric age [5]. Currently, there is a restricted body of evidence supporting therapies for neuronal regeneration following TBI [6]. Nevertheless, neurotrophins have shown a good potential to promote neurological recovery and enhance cognitive skills after TBI [6,7]. Among these, nerve growth factor (NGF) has emerged as the most promising in reducing TBI-induced excitotoxicity, limiting mitochondrial damage, promoting neoangiogenesis, and safeguarding oligodendrocytes [8]. There is both preclinical and clinical evidence which supports how treatment with NGF can positively impact the natural progression of clinical pictures secondary to TBI. NGF has a good ability to diffuse into the brain parenchyma via an anterior pathway through the olfactory nerve and a posterior pathway through the trigeminal nerve. Therefore, the intranasal route of NGF administration has received increasing attention in recent years due to its low invasiveness and ease of performance [9]. Remarkable results have been achieved with intranasal NGF administration in children with severe neurological damage in a variety of conditions such as meningitis, chronic vegetative state after cardiac arrest, and unresponsive wakefulness syndrome post-TBI [10,11,12,13]. In the light of these encouraging results, we report a case of a child with neurological sequelae after severe TBI who received intranasal administration of human-recombinant NGF (hr-NGF).

## 2. Case Presentation

A three-year-old boy was admitted to a Pediatric Emergency Department and then to Pediatric Intensive Care Unit for severe TBI, secondary to a car accident. He underwent intensive care and intracranial pressure monitoring for a basilar skull fracture involving the facial skeleton (mandible). Brain CT (computerized tomography) scan showed extra axial hematoma in the right fronto-parieto-temporal region, intraventricular hemorrhage, diffuse axonal injury and post traumatic pseudoaneurysm in internal carotid artery. It was necessary to place an external cerebrospinal fluid (CSF) shunt, which was later internalized. As a complication, he developed cerebral venous sinus thrombosis, which was treated with enoxaparin. Surgical reduction of the humeral fracture and osteosynthesis with titanium elastic nail (TEN) was performed. Prophylactic anticonvulsant therapy with levetiracetam was started.

After extubation, an unstable state of vigilance with chaotic environmental participation was observed. Neurological examination revealed hypertonia in the distal lower limbs, reducible (with tonic-clonic jerks present) and brisk deep tendon reflexes in the lower limbs. There was no clonus when evoked. Anisocoria with mydriasis in the left eye and reduced responsiveness to light stimuli were observed. Functionally, disorganized spontaneous movements of the upper limbs were observed; extensor jerks were visible in the lower limbs.

Auditory evoked potentials in acute phase on the right side were normal, while on the left side a poor definition of all components was observed, suggesting a primary alteration of the cochlea/ipsilateral cranial nerve VIII.

Somatosensory evoked potentials (SSEP) performed in acute phase showed presence of cortical N20 bilaterally, with reduced amplitude in response to left-sided stimulation; completely normal when repeated one month after the event.

Two months after the trauma, the child was admitted to the Neurorehabilitation Unit to receive intensive treatment.

At the time of admission, the child progressively improved his state of consciousness in a few days. He stabilized his functions more and more, with recovery of normal blood pressure, heart rate and respiratory function. Functional profile of double hemiparesis with major involvement of the left side was observed. The child was breathing spontaneously through tracheostomy.

The rehabilitation program was carried out before and after the treatment with NGF. Before NGF treatment, the child was admitted to the sub-intensive care unit at Bambino Gesù Pediatric Hospital in Palidoro (Rome) in a state of minimal responsiveness. He originally presented with severe left hemi-neglect, significant motor deficits, and a tracheostomy.

He exhibited no head/trunk control or ability to perform postural transitions. Oro-mandibular movements were limited, but he showed moderate visual tracking while in a supine position.

### 2.1. Initial Therapy Results

Following inpatient rehabilitation, a comprehensive and detailed assessment of cognitive, neuropsychological, academic and motor aspects was undertaken.

#### 2.1.1. Neurological Assessment

Therapy led to improvements in head control, with restored movement across all spatial planes, along with the presence of voluntary movements in the left leg, pelvis, and oro-buccal areas. Sitting posture (with and without postural support) was introduced, showing progressive improvement alongside enhanced spatial perception on the left side. This also marked the beginning of proximal movements in the ipsilateral limb, although distal sections remained entirely plegic. Trunk control improved to the extent of enabling verticalization using Knee–Ankle–Foot Orthosis (KAFO), followed by assisted walking with an Ankle–Foot Orthosis (AFO) walker.

Despite these advancements, the upper left limb remained predominantly inactive, showing only proximal shoulder movements when stimulated. As therapy progressed, significant advancements were noted in gross motor movements and assisted walking. Fear of falling diminished, static and dynamic balance improved, and autonomy increased across postural transitions. Standing endurance and posture compensation were reduced, even during ambulation.

#### 2.1.2. Behavioral and Cognitive Aspects

Social interaction with adults improved, though high frustration levels persisted. Verbal stereotypies, primarily imitative, were also observed. Social interaction with peers improved; he showed attraction toward them, reduced difficulty separating from parents, and enhanced attention, rule-following, and frustration tolerance.

#### 2.1.3. Fine Motor Coordination

Although the right upper limb was preferred, the child occasionally integrated bilateral use when prompted, demonstrating difficulty but achieving some support functionality. Contralateral elbow flexion and enhanced eye–hand coordination were observed, enabling pupillary convergence training for the right eye (severe divergent strabismus with ocular hypofunction). No assessments were conducted for the left upper limb due to its significant functional limitations, rendering standard evaluation scales inapplicable.

### 2.2. Intranasal Human-Recombinant NGF (hr-NGF) Administration

Nine months after the traumatic brain injury (TBI), due to persistent motor dysfunction, the possibility of initiating therapy with intranasal administration of human recombinant hr-NGF was considered. Written informed consent was obtained from the parents before beginning treatment. The study was approved by the Ethic Committee of the Fondazione Policlinico Universitario Agostino Gemelli—IRCCS in Rome, Italy (approval n. 5169/20, ID 2989) and is registered as a clinical trial (EudraCT number 2019-002282-35).

The child received hr-NGF (Cenegermin, manufactured by Dompè Farmaceutici, Milan, Italy) via intranasal administration in four cycles, each lasting seven days. The aqueous hr-NGF solution, administered at a total dose of 50 µg/kg, was designed to effectively stimulate NGF receptors, primarily Tyrosine receptor kinase A (TrkA), in key cholinergic and serotonergic regions of the brain, as supported by previous studies [14,15].

The used product was Cenegermin-bkbj (hr-NGF) as a 0.002% ophthalmic solution (20 µg/mL). The vials were stored at 4 °C until use. The total dose of hr-NGF was divided into four treatment cycles, with each cycle representing one-quarter of the total dose. This portion was further divided into 21 doses, administered 3 times daily over 7 consecutive days. The administration cycles commenced on days 1, 31, 61, and 91 of the treatment period. The dose and administration volume for each session were calculated based on the patient’s weight. The hr-NGF solution was delivered using the MAD Nasal™ (Intranasal Mucosal Atomization Device from Teleflex, model MAD100, version IPN048826), which sprays the solution in a fine mist (30 microns) to enhance absorption via olfactory and trigeminal nerve pathways, without requiring dilution or lyophilization. Before each NGF administration, the nostrils were cleaned with 1 mL of saline solution and gently suctioned to minimize any potential impact on drug absorption.

## 3. Results

### 3.1. Clinical Results After hr-NGF Treatment

#### 3.1.1. Motor Function

The child achieved balanced static and dynamic weight distribution on the lower limbs, with enhanced stability, endurance, and equilibrium reactions. Despite persistent postural compensations (e.g., marked hyperlordosis, leftward head rotation, and buccal synkinesis), spatial misperception during directional changes and fear of falling decreased. He became independent in both standing and walking, successfully walking unassisted over long distances without external support.

Although activities remained predominantly reliant on the right upper limb, occasional intentional movements with the left arm were observed, involving shoulder and elbow movements and, by irradiation, the hand. When a target was placed in his hand, grasping was performed using a raking motion (exploiting grasp reflex).

A gradual weaning from the tracheostomy was carried out without its removal, because during episodes of respiratory exacerbation it was needed.

#### 3.1.2. Language and Verbale Cognitive Skills

An assessment of language and verbal cognition was carried out approximately 8 months after TBI, at the end of inpatient rehabilitation. A more complete evaluation (language, cognitive and neuropsychological skills) was carried out after four cycles of intranasal NGF. Table 1 shows results obtained by the patient in the specific domains at baseline and follow-up.

In addition to the assessments already reported in Table 1, after NFG treatment evaluation, executive functioning was also investigated, through administration of the FE-PS 2–6; Italian Battery for the Evaluation of Executive Functions in Preschool Age (Usai et al., 2017) [22]. These findings are fully shown in Table 2.

#### 3.1.3. Electroencephalogram (EEG)

EEG performed at the beginning and at the end of the rehabilitation hospitalization showed asymmetrical background activity, better organized on the left, with numerous right anterior epileptiform abnormalities, tending to diffuse to the right hemisphere and the contralateral hemisphere. These abnormalities were markedly reduced on awakening.

EEG recording performed after one month of the last cycle of NGF treatment showed a significant reduction in epileptiform abnormalities.

#### 3.1.4. Neuroimaging

The first magnetic resonance imaging (MRI) study of the brain and spinal cord was performed four days after the head injury and eight months before the start of intranasal NGF administration. The second brain MRI was repeated one month after the end of treatment.

The first brain MRI showed an extensive extra-axial intracanalar hematoma partially occupying the posterior dorso-lumbar endocanalar region, resulting in anterior dislocation of the distal spinal cord tract; lumbar endocanalar hematoma partially mixed with the roots of the cauda; chamber-like appearance of the anterior perimedullary CSF space at the level of the cervico-dorsal tract. Brain examination revealed the following: a caliber asymmetry between the two internal carotid arteries due to a reduction in the caliber of the right one; subarachnoid hemorrhage, especially in the base cisterns and adjacent to the carotid bifurcation; numerous areas of restriction on diffusion-weighted imaging (DWI) involving both distal cortical areas and areas of deep white and gray matter; presence of bilateral fronto-temporal intracranial extra-axial fluid and a fluid-hematic layer along the outer profile of the left sigmoid transverse tract in the retromastoid area; and the presence of a hematic layer bilaterally in the retro-occipital region.

After one month from the last cycle with intranasal NGF administration, brain MRI with contrast showed neuroradiological findings substantially stable compared to previous examinations (in particular, the reduction in caliber of the internal carotid artery and of the right middle cerebral artery). There also remained a subtle opacification defect of non-occluding appearance at the level of the left transverse-sigmoid sinus, in likely outcome. No areas of diffusivity restriction of recent significance were evaluated. The morphology and amplitude of the ventricular system were basically unchanged; the amplitude of the subarachnoid spaces was also unchanged. The diffuse thickening of the bihemispheric and sub-tentorial dura was unchanged. The remaining MRI and Angio-MRI intracranial findings were also substantially unchanged.

### 3.2. Somatosensory-Evoked Potential (SSEP), Motor Evoked Potentials (MEPs), Auditory Brainstem Evoked Potentials (BAEPs)

Before the intranasal hr-NGF treatment and after one week from the brain injury evoked potentials (sensory only) were performed. Auditory brainstem evoked potentials (BAEPs) on the right side were normal. BAEPs on the left had poor definition of all components as for primary cochlea/VIII nerve alteration ipsilaterally. BAEPs were not evaluated after the treatment.

Upper limbs SSEP showed cortical N20 present bilaterally (with a reduced amplitude from left stimulus). Lower limbs SSEP identified a normal range bilaterally.

After one month from the last cycle with intranasal NGF administration, the upper limbs SSEP showed a normal bilateral representation of latency parameters, amplitude and morphology of the peripheral and cortical P25 components from right upper limb stimulation. Reduced amplitude of cortical P25 potential with normal latency from left upper limb stimulation were observed.

The lower limbs SSEP identified a cortical P40 potential not reproducible bilaterally.

Regarding the MEP, MEP of the upper limb showed a normal bilateral representation of the latency, amplitude, and morphology parameters of the peripheral and cortical components P25 from stimulation of the right upper limb. A reduction in the amplitude of the cortical P25 potential was observed, with normal latency from stimulation of the left upper limb.

## 4. Discussion

In this study, we present the outcomes of intranasal hr-NGF treatment administered to a three-year-old boy who sustained a diffuse axonal injury due to a severe TBI. Diffuse axonal injury (DAI) has become a hallmark of TBI injury. Most axonal damage does not derive from prompt mechanical disruption but rather from secondary injury mechanisms that can cause prolonged damage to axons after the initial event. This secondary injury involves a cascade of biochemical and cellular events that lead to further axonal degeneration, often exacerbated by several factors, such as inflammation and excitotoxicity, which can endure for days, weeks, or even months. Pediatric patients are at particular risk for shear stress lesions. This is due to the reduced myelin content and higher water percentage of the pediatric brain.

From a clinical perspective, functional outcomes after DAI vary widely, as some children have profound impairment while others recover better [23].

Although cardiopulmonary resuscitation procedures have been implemented, widely disseminated, and validated, severe outcomes of TBI have continued to be recorded in recent years. In fact, there is an unmet need for the development of innovative treatments and medications [24].

Rehabilitative care for TBI patients has resulted in only modest neurological improvement. Thus, our group decided to pioneer new therapeutic avenues by starting an experimental therapy with intranasal hr-NGF. There is a solid body of evidence in the literature showing the effect of NGF in a noteworthy decrease in neuroinflammation, protein aggregation, and mitochondrial dysfunction at the cellular and molecular levels, along with an enhancement of angiogenesis and protection of oligodendrocytes [15].

The inability to deliver neurotrophins directly to the CNS due to the blood–brain barrier has prevented significant progress towards effective treatment in the past. Delivery of NGF to the brain via the olfactory pathway has emerged as an innovative, non-invasive and safe way to achieve effective concentrations of NGF in targeted CNS regions [5]. In preclinical models, the intranasal administration of NGF has shown effective effects following severe TBI. These include specifically reducing TBI-induced reactive astrogliosis and neuroinflammation. Clinically, NGF has been shown to prevent the onset of motor coordination deficits and locomotor dysfunction following TBI in young rats [25]. Intranasal administration of NGF leads to significant concentrations of this neurotrophin in the brain, particularly in the frontal and parietal cortices, thalamus, cerebellum and striatum, which are most commonly affected by TBI. Only Young et al. found that intranasal NGF was not an effective treatment for functional motor recovery after TBI in rats [26]. In this study, NGF was administered intranasally for a shorter duration (only a single course of 7 days) than in the current study. Therefore, the effects of NGF may not have been fully developed compared to other studies due to this brief course of administration. Moreover, considering the preliminary nature of these findings, further clinical trials with a wide range of patients are needed to validate the applicability of hr-NGF for TBI in children.

As for therapy safety, actually, the currently available evidence in the literature is poor, even more in children, but a recent comprehensive review on this topic showed a substantial absence of reported adverse effects [15]. Currently, NGF therapy is expensive and only used for experimental purposes in limited clinical trials. The use of such a therapy in clinical trials with larger numbers of patients would provide new insights into efficacy and safety in the general pediatric population. Large-scale use as described above would also help to reduce costs to some extent.

In this specific case we reported, intranasal hr-NGF treatment significantly improved the functional outcome, as evidenced by the marked improvement in both clinical and neurological conditions.

From a motor perspective, the child demonstrated notable advancements, achieving independent standing and walking.

With regard to the behavioral aspects observed in the child during the first hospitalization and the significant improvements observed after the four cycles of NGF, it is necessary for the neuropsychological battery at the two assessments, as shown in Table 1, to be different. In fact, at the first assessment (before NGF treatment), the child needed external guidance to organize activities and maintain attention on the task, especially in the presence of distracting elements. His attention span was slightly reduced for his age and some oppositional attitudes were also observed in response to unpleasant activities, as well as the habit of engaging in escape behavior or perseverative attitudes. After NGF treatment, the child was more proactive and participative in activities and had greater communicative initiative. There was also a reduction in oppositional and perseverative behavior. These improvements made it possible to carry out a more comprehensive developmental assessment.

Analyzing the results of cognitive assessments, it is possible to point out, as already mentioned, the possibility of proceeding with a more in-depth evaluation, taking into account all the sub-areas studied. Good skills are confirmed in the area of verbal comprehension, a strong point of the child’s profile. In the evaluation following cycles of intranasal NGF, a global profile within the expected range is observed, characterized by normal skills in working memory; skills within the expected range in fluid reasoning; and skills below the expected range in visuospatial reasoning and processing speed. In terms of linguistic skills, adequate morphosyntactic comprehension and lexical production are observed, but age-related deficiencies in narrative skills. Furthermore, adequate skills were highlighted in both visual–motor integration (unchanged between the two assessments) and visual perception. From the battery for the evaluation of executive functions, skills in line with age expectations in inhibition ability were observed in all the areas investigated, with the exception of a slight discrepancy in two tests investigating inhibition in motor response, where the child seems to show greater skills when the task is continuous over time, as shown in Table 2.

Focusing on the electroencephalographic evaluation, there was a significant improvement as the detected epileptiform activity was less detectable after NGF administration. The neuroimaging evaluations performed showed substantial stability.

Certainly, some neurological improvements have been observed. Determining the true nature of these changes is quite difficult, as they may be partly related to the natural physiological recovery that occurs after TBI. In fact, a number of adaptive mechanisms kick in immediately after the insult, leading to neuroplastic recovery, which helps to restore certain neurological functions.

Oligodendrogenesis, the generation of new oligodendrocytes, is largely responsible for recovery after TBI [27]. Oligodendrocytes play a key role in the brain, forming a myelin sheath around axons and restoring a rich neural network when the main circuit is damaged [28]. The interaction between axons and oligodendrocytes regulates the speed of signal transmission, in a way that is activity-dependent, highlighting the importance of oligodendrocytes in enhancing adaptability. In this setting, undifferentiated precursors emerge from the quiescent state. They proliferate, migrate and mature into myelinating oligodendrocytes [28]. Therefore, in terms of neuroplasticity and signal propagation, they are able to compensate for much of the damaged function.

Hence, it is likely that the neurological improvement observed in our patient is partly due to this inherent recuperative mechanism, although assessing its extent would be difficult [29].

Given the remarkable clinical improvement in such a relatively brief timeframe, we hypothesize that most of the ameliorations we observed were due to treatment with NGF.

In recent decades, NGF has become increasingly important in medical research due to its critical role in regulating the connectivity network and differentiation of neurons in both the peripheral and central nervous systems.

Chiaretti et al. demonstrated the crucial roles of NGF in neurogenesis and neuronal repair. Their study measured NGF levels in the cerebrospinal fluid of children with TBI at two time points: T1 (2 h post-injury) and T2 (48 h post-injury). The findings revealed that children who exhibited better outcomes had higher NGF levels compared to those with poorer outcomes [30].

This was followed by intriguing data from an experimental study in rats using intranasal NGF in a TBI model, which showed remarkable progress in motor dysfunction and regulation of neuroinflammation [25].

In view of the brilliant results obtained, our group decided to perform a trial to evaluate the clinical and neuroradiologic improvement by intranasal administration of NGF in children with brain damage. The study was conducted in accordance with the Declaration of Helsinki and approved by the Ethics Committee (approval n° 5169/20, ID 2989) of the Fondazione Policlinico Universitario Agostino Gemelli—IRCCS, Rome (Italy). Written informed consent was obtained by the child’s parents. Since then, remarkable results have been obtained in a clinical scenario where no other therapeutic strategies are currently available [11].

Our team has conducted further investigations into the effects of intranasal hr-NGF on individuals suffering from significant brain damage due to severe brain injuries. These studies have primarily revealed enhancements in motor functions, marked by a significant reduction in muscle hypertonicity and spasticity. This underscores the vital role of NGF in addressing striatal cholinergic dysfunction [10,11,24].

In the case we reported, we observed a substantial steady state through neuroimaging and a significant gain in cognitive abilities.

We would like to emphasize that, on a qualitative level, both the child and the caregivers have reported a significant overall improvement in performance and responsiveness in daily life.

## 5. Conclusions

The use of intranasal hr-NGF appears to be a viable option for treating children who experience neurological complications following TBI. Our findings are encouraging and may lay the groundwork for future clinical trials focused on assessing the effectiveness of intranasal hr-NGF in enhancing cognitive abilities and overall health outcomes in children suffering from diffuse axonal injury. This could mark the beginning of a broader clinical initiative aimed at evaluating how intranasal NGF can improve neurological recovery and functional capabilities in young patients with severe TBI.

Although single case studies can offer valuable insights, particularly for rare conditions or unique treatment responses, and may provide useful information or generate hypotheses, their limitations highlight the importance of caution when attempting to generalize the results. Larger, controlled studies, such as randomized clinical trials, are necessary to validate the findings and establish evidence-based practices.

The preliminary results presented here, combined with the straightforward method of drug administration, warrant further investigation, particularly in TBI patients who initially exhibit more favorable neurological conditions. This will help clarify the benefits of NGF for brain function recovery. Although additional controlled, randomized, double-blind studies are necessary to elucidate the neuroprotective effects of this neurotrophin, intranasal NGF shows great promise as a therapeutic option for children facing neurological challenges after significant head injuries.

## Figures and Tables

**Table 1:** Specific domain results at baseline and follow-up.

Task	Skills Investigated	Domains	Scores Pre NGF Treatment	Scores Post NGF Treatment
Wechsler Preschool and Primary Scale of Intelligence—fourth edition (WPPSI-IV; Wechsler, 2012; It Trasl: Saggino et al., 2019) [16]	Cognitive Functioning	Full Scale Intelligence Quotient (FSIQ; M = 100 ± 15)	Unavailable	80
		Verbal Comprehension Index (VCI; M = 100 ± 15)	127	111
		Visual Spatial Index (VSI; M = 100 ± 15)	Unavailable	67
		Fluid Reasoning Index (FRI; M = 100 ± 15)	Unavailable	81
		Working Memory Index (WMI; M = 100 ± 15)	Unavailable	89
		Processing Speed Index (PSI; M = 100 ± 15)	Unavailable	66
Boston Naming Test (Kaplan et al., 1983) [17]	Language ability	Visual confrontation naming/lexical production	+0.22 sd	unavailable
Language Comprehension Assessment Tests (PVCL; Lancaster et al., 2003) [18]		Morphosyntactic comprehension	58.4 (medium-low average profile for age)	53.4 (medium-low average profile for age)
Phono Lexical Test (TFL; Marotta et al.; 2007) [19]		Receptive language/lexical comprehension	unavailable	25th percentile
		Expressive language/lexical production	unavailable	75th–90th percentile
Bus story test (Renfrew; 1997; It Transl. 2015) [20]		Narrative speech	Unavailable	−2 sd
Developmental Test of Visual-Motor Integration (VMI; Beery and Buktenica, 1996) [21]	Coordination of visual and motor skills	Visual Motor Integration	SS 92	SS 90
		Visual Perception	Unavailable	SS 87

**Table 2:** FE-PS 2–6; Italian Battery for the Evaluation of Executive Functions in Preschool Age.

Tasks	Domains	Scores
Draw a circle	Inhibition of Continuous Motor Response	50th percentile
Stroop day and night	Inhibition of verbal response	Accuracy 75th percentile Time 50th percentile
The elephant and the bear	Inhibition of motor response	10th percentile
Compare the figures	Control of impulsive response and working memory	Time 90th percentile Errors 75th percentile
The fish game	Interference management	Accuracy 50th/75th percentile Time < 5th percentile
The gift	Inhibition of impulsive behavior	50th/75th percentile
The color and shape game	Inhibition and working memory	25th/50th percentile
Keep in mind	Updating working memory	75th/90th percentile

## Data Availability

The raw data supporting the conclusions of this article will be made available by the authors on request.
